# Supplementary material for: MetaQTL: a package of new computational methods for the meta-analysis of QTL mapping experiments
Source: BMC Bioinformatics. 2007 Feb 8;8:49. doi: 10.1186/1471-2105-8-49 (PMC1808479; doi:10.1186/1471-2105-8-49)
Supplement: Additional File 7 — MetaQTL Package : jar file and tutorial. This Zip archive contains both the MetaQTL JAR file and the files of the tutorial. [file 1471-2105-8-49-S7.zip › org.inra.metaqtl/doc/org/metaqtl/util/OffspringManager.HaldaneWaddingtonProportion.html]

OffspringManager.HaldaneWaddingtonProportion


|  |  |  |
| --- | --- | --- |
| **Overview** \| **Package** \| **Class** \| **Use** \| **Tree** \| **Deprecated** \| **Index** \| **Help** | |  |
| **PREV CLASS**   NEXT CLASS | **FRAMES**    **NO FRAMES**     **All Classes** |  |
| SUMMARY: NESTED \| FIELD \| CONSTR \| METHOD | DETAIL: FIELD \| CONSTR \| METHOD |  |


---


## org.metaqtl.util Class OffspringManager.HaldaneWaddingtonProportion

```
java.lang.Object
  org.metaqtl.util.OffspringManager.HaldaneWaddingtonProportion
```

**Enclosing class:**: OffspringManager

---

``` public class OffspringManager.HaldaneWaddingtonProportion extends java.lang.Object ```

---

| **Field Summary** | |
| --- | --- |
| `double` | `C` |
| `static double` | `C_coeff` |
| `double` | `D` |
| `static double` | `D_coeff` |
| `double` | `dC` |
| `double` | `dD` |
| `double` | `dE` |
| `double` | `dF` |
| `double` | `dG` |
| `double` | `E` |
| `static double` | `E_coeff` |
| `double` | `F` |
| `static double` | `F_coeff` |
| `double` | `G` |
| `static double` | `G_coeff` |


**Constructor Summary**

`OffspringManager.HaldaneWaddingtonProportion()`


**Method Summary**

**Methods inherited from class java.lang.Object**

`clone, equals, finalize, getClass, hashCode, notify, notifyAll, toString, wait, wait, wait`

**Field Detail**

### C\_coeff

```
public static final double C_coeff
```

**See Also:**: Constant Field Values

---


### D\_coeff

```
public static final double D_coeff
```

**See Also:**: Constant Field Values

---


### E\_coeff

```
public static final double E_coeff
```

**See Also:**: Constant Field Values

---


### F\_coeff

```
public static final double F_coeff
```

**See Also:**: Constant Field Values

---


### G\_coeff

```
public static final double G_coeff
```

**See Also:**: Constant Field Values

---


### C

```
public double C
```

---


### D

```
public double D
```

---


### E

```
public double E
```

---


### F

```
public double F
```

---


### G

```
public double G
```

---


### dC

```
public double dC
```

---


### dD

```
public double dD
```

---


### dE

```
public double dE
```

---


### dF

```
public double dF
```

---


### dG

```
public double dG
```


**Constructor Detail**

### OffspringManager.HaldaneWaddingtonProportion

```
public OffspringManager.HaldaneWaddingtonProportion()
```


---


|  |  |  |
| --- | --- | --- |
| **Overview** \| **Package** \| **Class** \| **Use** \| **Tree** \| **Deprecated** \| **Index** \| **Help** | |  |
| **PREV CLASS**   NEXT CLASS | **FRAMES**    **NO FRAMES**     **All Classes** |  |
| SUMMARY: NESTED \| FIELD \| CONSTR \| METHOD | DETAIL: FIELD \| CONSTR \| METHOD |  |


---
